# Does reconstruction affect outcomes following exclusively endoscopic endonasal resection of benign orbital tumors: A systematic review with meta‐analysis

**DOI:** 10.1002/wjo2.13

**Published:** 2022-03-31

**Authors:** Ashton E. Lehmann, Manuela von Sneidern, Sarek A. Shen, Ian M. Humphreys, Waleed M. Abuzeid, Aria Jafari

**Affiliations:** ^1^ Department of Otolaryngology‐Head and Neck Surgery Vanderbilt University Medical Center Nashville Tennessee USA; ^2^ Department of Otolaryngology‐Head and Neck Surgery Geisinger Medical Center Danville Pennsylvania USA; ^3^ Department of Otolaryngology‐Head and Neck Surgery New York University School of Medicine New York New York USA; ^4^ Department of Otolaryngology‐Head and Neck Surgery Johns Hopkins Hospital Baltimore Maryland USA; ^5^ Division of Rhinology and Endoscopic Skull Base Surgery, Department of Otolaryngology‐Head and Neck Surgery University of Washington Seattle Washington USA

**Keywords:** cavernous hemangioma, diplopia, endoscopic endonasal surgery, enophthalmos, meningioma, orbital reconstruction, orbital tumor, outcomes, schwannoma

## Abstract

**Objective:**

As exclusively endoscopic endonasal resection of benign orbital tumors has become more widespread, high‐quality outcomes data are lacking regarding the decision of when and how to reconstruct the medial orbital wall following resection. The goal of this study was to systematically review pertinent literature to assess clinical outcomes relative to orbital reconstruction practices.

**Methods:**

Data Sources: PubMed, EMBASE, Web of Science. A systematic review of studies reporting exclusively endoscopic endonasal resections of benign orbital tumors was conducted. Articles not reporting orbital reconstruction details were excluded. Patient and tumor characteristics, operative details, and outcomes were recorded. Variables were compared using *χ* ^2^, Fisher's exact, and independent *t* tests.

**Results:**

Of 60 patients included from 24 studies, 34 (56.7%) underwent orbital reconstruction following resection. The most common types of reconstruction were pedicled flaps (*n* = 15, 44.1%) and free mucosal grafts (*n* = 11, 32.4%). Rigid reconstruction was uncommon (*n* = 3, 8.8%). Performance of orbital reconstruction was associated with preoperative vision compromise (*p* < 0.01). The tendency to forego orbital reconstruction was associated with preoperative proptosis (*p* < 0.001), larger tumor size (*p* = 0.001), and operative exposure of orbital fat (*p* < 0.001) and extraocular muscle (*p* = 0.035). There were no statistically significant differences between the reconstruction and nonreconstruction groups in terms of short‐ or long‐term outcomes when considering all patients. In patients with intraconal tumors, however, there was a higher rate of short‐term postoperative diplopia when reconstruction was foregone (*p* = 0.041). This potential benefit of reconstruction did not persist: At an average of two years postoperatively, all patients for whom reconstruction was foregone either had improved or unchanged diplopia.

**Conclusion:**

Most outcomes assessed did not appear affected by orbital reconstruction status. This general equivalence may suggest that orbital reconstruction is not a necessity in these cases or that the decision to reconstruct was well‐selected by surgeons in the reported cases included in this systematic review.

## Highlights

- Question: Does orbital reconstruction affect outcomes following exclusively endoscopic endonasal resection of benign orbital tumors?
- Findings: In this systematic review and meta‐analysis of 60 patients included from 24 studies, there were no statistically significant differences between the reconstruction (*n* = 34, 56.7%) and non‐reconstruction groups in terms of short‐ or long‐term outcomes when considering all patients. In patients with intraconal tumors, however, there was a higher rate of short‐term (<2 years) post‐operative diplopia when reconstruction was foregone (*p* = 0.041).
- Meaning: Most outcomes assessed did not appear affected by orbital reconstruction status. This general equivalence may suggest that orbital reconstruction is not a necessity in these cases or that the decision to reconstruct was well‐selected by surgeons in the reported cases included in this systematic review.

## Graphical abstract

In this systematic review and meta‐analysis of 24 studies reporting exclusively endoscopic endonasal resections of benign orbital tumors, there were no significant difference between the reconstruction and non‐reconstruction groups in terms of short‐ or long‐term outcomes when considering all 60 patients. In the patients with intraconal tumors (*n* = 43), however, there was a higher rate of short‐term post‐operative diplopia when reconstruction was foregone.

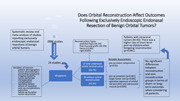

**Abbreviations**
BOT: benign orbital tumors
CHEER: Cavernous Hemangioma Exclusively Endonasal Resection
EER: exclusively endonasal resection
EOM: extraocular muscle

## INTRODUCTION

With recent innovations in skull base and sinonasal surgery, the use of exclusively endonasal resection (EER) for orbital tumors is increasing.^1^, ^2^, ^3^, ^4^ This is especially the case for benign orbital tumors (BOTs), which are commonly encapsulated and are, thus, more amenable to EER.^4^, ^5^ Compared to external approaches, the transnasal approach to EER of BOTs provides enhanced visualization and illumination while limiting postoperative morbidity by minimizing intraoperative retraction and trauma to surrounding structures.^5^, ^6^ The EER approach may, however, necessitate removal of large portions of orbital bone and periorbita, significant extraocular muscle (EOM) retraction, and intranasal exposure of intraconal structures,^6^, ^7^—all of which may contribute to untoward postoperative morbidity.^7^, ^8^ Indeed, a recent multicenter review of endoscopic orbital tumor resections reported that approximately one‐fifth of patients developed new‐onset enophthalmos and diplopia postoperatively.^3^ In response to such reports of postoperative morbidity, increasing attention has been paid to consideration of postresection reconstruction geared at minimizing these esthetic (i.e., enophthalmos) and functional (i.e., diplopia) issues.^8^

Advocates of reconstruction suggest that medial orbital wall reconstruction counterbalances bony removal and periorbita opening which may otherwise lead to unwanted enophthalmos and diplopia. While in practice it appears that most high‐volume endoscopic orbital surgeons do not routinely reconstruct following endoscopic transnasal orbital tumor removal (with an overall rate of reconstruction of 26.1% of reported cases),^3^ reconstructive decisions following EER are highly contested.^9^ Some surgeons suggest medial wall reconstruction to minimize diplopia may generally not be warranted for most patients.^10^ Others have posited that orbital reconstruction is frequently required to mitigate postoperative morbidity.^7^ Some have even suggested that reconstruction only be foregone if it is unfeasible or would jeopardize oncologic follow‐up.^8^

Given the rarity of orbital tumors and the relative nascency of the EER approach, high‐quality outcomes data are generally limited and are especially lacking in regard to the decision of when and how to reconstruct following EER.^4^, ^11^ Due to the predominance of case reports and case series in the relevant literature, a systematic review offers a robust approach to assess how orbital reconstruction affects clinical outcomes following EER for BOTs.^4^, ^5^ In the present analysis, we sought to systematically review and analyze the current literature to characterize the perioperative factors and clinical outcomes following exclusively endoscopic endonasal resections of benign primary orbital tumors in light of reported orbital reconstruction practices from individually extracted and aggregated data.

## METHODS

### Search strategy and selection criteria

A systematic review of studies reporting exclusively endoscopic endonasal resections of primary BOTs was performed in accordance with the Preferred Reporting Items for Systematic Reviews and Meta‐Analyses guidelines as previously described by Jafari et al.^4^ The results from the original search were uploaded to Covidence systematic review software (Covidence) for independent screening, multiphase review, and data extraction. Studies that did not include reporting of at least one EER for a BOT, abstracts without available full texts, review articles, cases of malignant tumors or metastases (as such lesions often have different operative characteristics and varying surgical goals), nonexclusively endoscopic cases, non‐English language articles, and articles not reporting reconstruction status were excluded. A final list of articles was then generated for data collection after the resolution of any conflicts by author consensus.

### Data collection

Variables of interest from the included articles were collected using a standardized worksheet. These variables included: (1) patient demographics, (2) tumor characteristics, (3) presenting symptoms, (4) imaging modality, (5) tumor pathology, (6) intraoperative complications, (7) operative details, (8) extent of resection, (9) short‐term postoperative complications (defined as within 14 days following surgery), (10) clinical outcomes (compared to each individual patient's preoperative presentation), (11) follow‐up duration, and (12) tumor recurrence. Of note, orbital fat exposure was defined based upon the mention of this occurrence during resection by the author(s) or by definition if a tumor was located in the intraconal space. EOM exposure was defined based upon muscle exposure seen in images/figures provided in the report or based upon mention of EOM exposure or retraction during resection by the author(s).

The previously developed Cavernous Hemangioma Exclusively Endonasal Resection (CHEER) system^11^ (the application of which has been broadened to other BOTs as well)^4^ was used to assign a stage to each tumor based on the imaging and location reported by the author(s) of each study. If there were insufficient data to determine the location of the tumor, these data were excluded for that tumor. The finalized extracted data set was reviewed by the authors (A. E. L. and A. J.) for accuracy.

### Statistical analysis

Individual participant data were compiled for analysis using the Patient, Problem or Population, Intervention, Comparison, Control of Comparator, Outcome framework. Results inclusive of patients with or without reconstruction were initially reported, and then stratified by orbital reconstruction status (i.e., patients who underwent reconstruction versus patients for whom reconstruction was foregone). Comparisons of clinical and demographic variables were completed using *χ* ^2^ statistics or Fisher's exact tests for binary and categorical variables when appropriate, and independent *t*‐tests for continuous variables. Statistical analyses were performed using Microsoft Excel (Microsoft Corp.) and Stata/SE 14 (StataCorp LP). All statistical tests were two‐tailed. The *α* level was set at <0.05 for statistical significance.

## RESULTS

**Figure 1:**
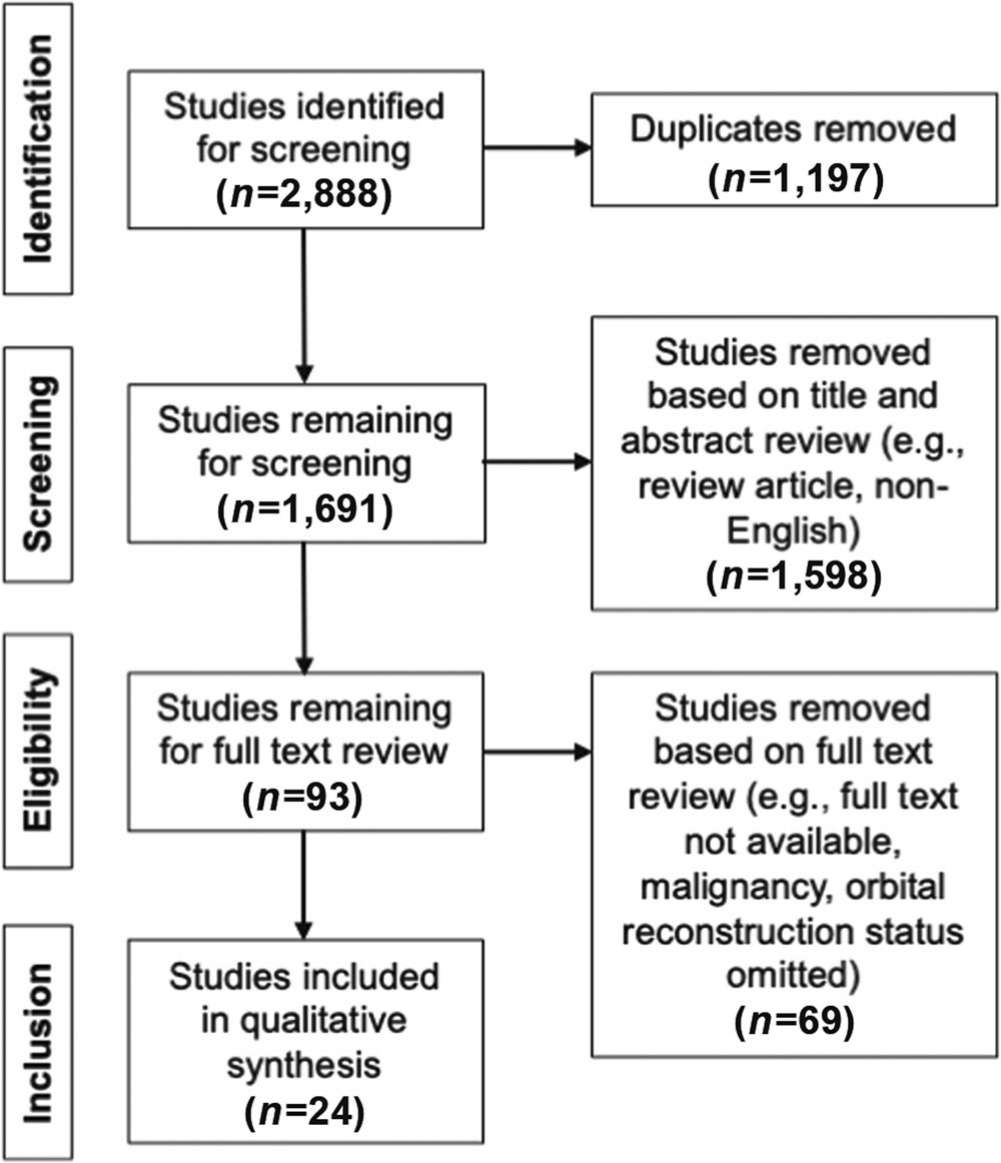
Article selection process for the systematic literature search. Systematic literature search selection process modified from that previously reported by Jafari et al.^4^

The previously conducted search strategy^4^ identified two 888 articles. After the removal of duplicates, one 691 unique titles and abstracts were screened. Ninety‐three full‐text articles were then assessed for eligibility based on the initial inclusion criteria, and 24 were ultimately included for data extraction and analysis. The 69 excluded articles included 57 that did not meet inclusion criteria and 12 that were omitted because they did not report orbital reconstruction status. Details of the selection process are shown in Figure 1.

**Figure 2:**
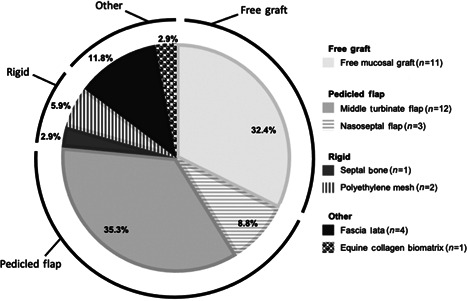
Distribution of types of medial orbital reconstruction reported. Pie chart of distribution of types of medial orbital reconstruction following exclusively endoscopic resection of benign orbital tumors (*N* = 34)

Twenty‐four studies inclusive of 60 patients who underwent exclusively endoscopic endonasal resections of 60 BOTs were included in the present analysis. Of these 60 patients, 34 (56.7%) underwent orbital reconstruction. The most common type of reconstructive technique involved pedicled flaps (*n* = 15, 44.1%), which included both nasoseptal (*n* = 3, 8.8%) and middle turbinate flaps (*n* = 12, 35.3%), followed by free mucosal grafts (*n* = 11, 32.4%; Figure 2). Rigid reconstruction (*n* = 3, 8.8%) was performed with either polyethylene mesh (*n* = 2, 5.9%) or septal bone (*n* = 1, 2.9%; Figure 2). Other reconstructive techniques utilized fascia lata (*n* = 4, 11.8%) and an equine collagen biomatrix (*n* = 1, 2.9%; Figure 2).

### Patient demographics and preoperative characteristics

Patient demographics and baseline preoperative characteristics for all BOTs and by reconstruction status are shown in Table 1. The mean patient age was (47.8 ± 16.0) years. The reported orbital tumors tended to be located in the intraconal compartment (*n* = 43, 71.7%), and the majority of patients who underwent orbital reconstruction had intraconal tumors (79.4% vs. 20.6% for extraconal tumors). In line with this, patients who underwent reconstruction trended toward higher CHEER stages, whereas patients for whom reconstruction was foregone trended toward lower CHEER stages, although these trends did not reach statistical significance (*p* = 0.360). Foregoing reconstruction was associated with a larger average tumor size (*p* < 0.001).

**Table 1:** Preoperative characteristics (*n* [%])

Item	All BOTs (*n* = 60)	Orbital reconstruction (*n* = 34)	No orbital reconstruction (*n* = 26)	*p* value
Demographics^a^				
Age (years, mean ± SD)	47.8 ± 16.0	46.6 ± 14.7	49.8 ± 18.1	0.470
Patient gender (*n* [%])				
Female	28 (46.7)	15 (44.1)	13 (50.0)	
Male	30 (50.0)	19 (55.9)	11 (42.3)	0.450
Not specified	2 (3.3)	0	2 (7.7)	
Tumor laterality (*n* [%])				
Left side	30 (50.0)	17 (50.0)	13 (50.0)	
Right side	14 (23.3)	6 (17.6)	8 (30.8)	0.360
Not specified	16 (26.7)	11 (32.4)	5 (19.2)	
Tumor size (mm, mean ± SD)^b^	14.7 ± 8.1	10.5 ± 7.3	19.0 ± 7.3	<0.001
Compartment (*n* [%])				
Intraconal	43 (71.7)	27 (79.4)	16 (50.0)	0.150
Extraconal	17 (28.3)	7 (20.6)	10 (50.0)	
CHEER stage (*n *[%])				
Ⅰ	17 (28.3)	7 (20.6)	10 (38.5)	0.090
Ⅱ/Ⅲ	11 (18.3)	5 (14.7)	6 (23.1)	
ⅣA	5 (8.3)	5 (14.7)	0	
ⅣB	27 (45.0)	17 (50.0)	10 (38.5)	
Presenting symptom (*n* [%])				
Visual field defect	23 (38.3)	21 (61.8)	2 (7.7)	<0.001
Decreased visual acuity	34 (56.7)	25 (73.5)	9 (34.6)	0.003
Diplopia	16 (26.7)	4 (11.8)	12 (46.2)	0.160
Proptosis	24 (40.0)	6 (17.6)	18 (69.2)	<0.001
Pain/headache	11 (18.3)	7 (20.6)	4 (15.4)	0.850
Color vision deficit	14 (23.3)	14 (41.2)	0	<0.001
Other symptoms	8 (13.3)	6 (17.6)	2 (7.7)	0.450

*Note*: NB: all “not specified” removed from statistical analysis.

Abbreviations: BOTs, benign orbital tumors; CHEER, Cavernous Hemangioma Exclusively Endonasal Resection.

^a^ Demographics, all BOTs (*n* = 55), orbital reconstruction (*n* = 34), no orbital reconstruction (*n* = 21).

^b^ Tumor size, all BOTs (*n* = 49), orbital reconstruction (*n* = 24), no orbital reconstruction (*n* = 25).

Decreased visual acuity (*n* = 34, 56.7%) and proptosis (*n* = 24, 40.0%) were the most common presenting symptoms. Greater percentages of patients who underwent reconstruction presented with visual field defects (61.8% vs. 7.7%; *p* < 0.001), decreased visual acuity (73.5% vs. 34.6%; *p* = 0.003), and color vision deficits (41.2% vs. 0%; *p* < 0.001) relative to those percentages of patients for whom reconstruction was foregone. Patients for whom reconstruction was foregone were more likely to present with proptosis relative to patients who underwent reconstruction (69.2% vs. 17.6%; *p* < 0.001).

Patients most commonly underwent both computed tomography and magnetic resonance imaging preoperatively (*n* = 42, 70.0%). There were no statistically significant associations with reconstruction status in regard to patient age or gender, tumor laterality, presentation with diplopia or pain/headache, or preoperative imaging modality used.

### Intraoperative characteristics

Intraoperative characteristics for all BOTs and by reconstruction status are shown in Table 2. Although the sample size for reports of number of hands used during resection was limited, there was a significant difference in the distribution of number of hands used in resections with or without reconstruction: Patients for whom reconstruction was foregone tended to have fewer hands used during resection (*p* = 0.02). Patients for whom reconstruction was foregone were also more likely to have only one nostril used for surgical access (53.8% vs. 11.8%; *p* = 0.01).

**Table 2:** Intraoperative characteristics

Item	All BOTs (*n* = 60)	Orbital reconstruction (*n* = 34)	No orbital reconstruction (*n* = 26)	*p* value
No. of hands (*n* [%])				
2	8 (13.3)	2 (5.9)	6 (23.1)	0.02
3	7 (11.7)	0	7 (26.9)	
4	6 (10.0)	4 (11.8)	2 (7.7)	
Not specified	39 (65.0)	28 (82.4)	11 (42.3)	
No. of nostrils (*n* [%])				
1	18 (30.0)	4 (11.8)	14 (53.8)	0.01
2	6 (10.0)	5 (14.7)	1 (3.8)	
Not specified	36 (60.0)	25 (73.5)	11 (42.3)	
Tumor pathology (*n* [%])				
Orbital cavernous hemangioma	48 (80.0)	26 (76.5)	22 (84.6)	0.360
Schwannoma	4 (6.7)	3 (8.8)	1 (3.8)	
Meningioma	3 (5.0)	3 (8.8)	0	
Solitary fibrous tumor	4 (6.7)	2 (5.9)	2 (7.7)	
Cyst	1 (1.7)	0	1 (3.8)	
Orbital fat exposure (*n* [%])				
Yes	19 (31.7)	8 (23.5)	11 (42.3)	<0.001
No	20 (33.3)	20 (58.8)	0	
Not specified	21 (35.0)	6 (17.6)	15 (57.7)	
EOM exposure (*n* [%])				
Yes	21 (35.0)	13 (38.2)	8 (30.8)	
No	19 (31.7)	18 (52.9)	1 (3.8)	0.035
Not specified	20 (33.3)	3 (8.8)	17 (65.4)	
Nasal packing (*n* [%])				
Yes	18 (30.0)	10 (29.4)	8 (30.8)	0.860
No	25 (41.7)	12 (35.3)	13 (50.0)	
Not specified	17 (28.3)	12 (35.3)	5 (19.2)	
Intra‐op complications (*n* [%])				
Bleeding	2 (3.3)	2 (5.9)	0	0.632
None	44 (73.3)	25 (73.5)	19 (73.1)	
Not specified	14 (23.3)	7 (20.6)	7 (26.9)	
Extent of resection (*n* [%])				
Complete/gross total	49 (81.7)	26 (76.5)	23 (88.5)	1.000
Subtotal	7 (11.7)	4 (11.8)	3 (11.5)	
Not specified	4 (6.7)	4 (11.8)	0	

*Note*: NB: all “not specified” removed from statistical analysis.

Abbreviations: BOTs, benign orbital tumors; EOM, extraocular muscle.

Patients without orbital reconstruction were more likely to have orbital fat exposed (*p* < 0.001). Of the patients without reconstruction for whom the presence or absence of EOM exposure was reported, those for whom reconstruction was foregone were more likely to have EOM exposure (*p* = 0.035). There were no other differences in intraoperative characteristics between patients with or without reconstruction. The majority of patients underwent either complete or gross total tumor resection (*n* = 49, 81.7%). Intraoperative complications were rare with only two cases of intraoperative epistaxis reported (3.3%).

### Clinical outcomes

Postoperative complications and clinical outcomes for all BOTs and by reconstruction status are shown in Table 3. There were no statistically significant differences between the reconstruction and nonreconstruction groups for all BOTs in terms of short‐term postoperative complications or longer‐term outcomes regarding visual acuity, visual field defects, color vision, diplopia, proptosis, enophthalmos, pain/headache, or tumor recurrence. However, when considering only intraconal tumors (see Table 4), a significantly greater proportion of patients for whom reconstruction was foregone experienced short‐term, postoperative diplopia (31.3%) compared to those who underwent reconstruction (7.4%; *p* = 0.041). A similar trend was seen when assessing both intraconal and extraconal tumors together: Approximately a quarter (23.1%) of patients for whom reconstruction was foregone had short‐term, postoperative diplopia, whereas only 8.8% of patients who underwent reconstruction demonstrated new or worsening diplopia postoperatively (*p* = 0.13). These findings, however, did not appear to persist into longer‐term follow‐up (with an average follow‐up duration of approximately 2 years), such that 100% of patients for whom reconstruction was foregone either had improved or unchanged diplopia at follow‐up.

**Table 3:** Postoperative complications and outcomes

Item	All BOTs ± reconstruction (*n* = 60)		Orbital reconstruction (*n* = 34)		No orbital reconstruction (*n* = 26)		*p* value
Short‐term post‐op complications (<2 weeks) (*n* [%])							
Visual acuity defect	2 (3.3)		1 (2.9)		1 (3.8)		1.00
Diplopia	9 (15.0)		3 (8.8)		6 (23.1)		0.13
Cranial nerve palsy	5 (8.3)		4 (11.8)		1 (3.8)		0.27
Epistaxis	3 (5.0)		3 (8.8)		0		0.25
Infection	1 (1.7)		0		1 (3.8)		0.43
Other^a^	5 (8.3)		5 (14.7)		0		n/a
None	40 (66.7)		21 (61.8)		19 (73.1)		0.51
Not specified	1 (1.7)		1 (2.9)		0		n/a
Length of follow up (months, mean ± SD)^b^	15.0 ± 17.1		10.4 ± 8.0		23.0 ± 23.0		
Visual acuity (*n* [%])^c^							
Improved/no change	33 (97.1)		24 (96.0)		9 (100.0)		1.00
Worse	1 (2.9)		1 (4.0)		0		
Visual field (*n* [%])^d^							
Improved/no change	22 (100.0)		21 (100.0)		1 (100.0)		n/a
Worse	0		0		0		
Color vision (*n* [%])^e^							
Improved/no change	14 (100.0)		14 (100.0)		0 (0.0)		n/a
Worse	0		0		0		
Diplopia (*n* [%])^f^							
Improved/no change	14 (93.3)		3 (75.0)		11 (100.0)		0.26
Worse	1 (6.7)		1 (25.0)		0		
Proptosis (*n* [%])^g^							
Improved/no change	23 (100.0)		6 (100.0)		17 (100.0)		n/a
Worse	0		0		0		
Enophthalmos	5 (8.3)		3 (8.8)		2 (7.7)		0.87
Pain/headache (*n* [%])^h^							
Improved/no change	8 (100.0)		5 (100.0)		3 (100.0)		n/a
Worse	0		0		0		
Tumor recurrence (*n* ([%])							
Yes	2 (3.3)		2 (5.9)		0		1.00
No	37 (61.7)		28 (82.4)		9 (34.6)		
Not specified	21 (35.0)		4 (11.8)		17 (65.4)		

*Note*: NB: all “not specified” removed from statistical analysis.

Abbreviation: BOTs, benign orbital tumors.

^a^ Other short‐term postoperative complications included dyschromatopsia, palpebral edema, cerebrospinal fluid rhinorrhea, hyposmia, and frontal hypesthesia.

^b^ Length of follow up, all BOTs ± reconstruction (*n* = 55), orbital reconstruction (*n* = 32), no orbital reconstruction (*n* = 23).

^c^ Visual acuity, all BOTs ± reconstruction (*n *= 34), orbital reconstruction (*n* = 25), no orbital reconstruction (*n* = 9).

^d^ Visual field, all BOTs ± reconstruction (*n* = 22), orbital reconstruction (*n* = 21), no orbital reconstruction (*n* = 1).

^e^ Color vision, all BOTs ± reconstruction (*n *= 14), orbital reconstruction (*n* = 14), no orbital reconstruction (*n* = 0).

^f^ Diplopia, all BOTs ± reconstruction (*n* = 15), orbital reconstruction (*n* = 4), no orbital reconstruction (*n* = 11).

^g^ Proptosis, all BOTs ± reconstruction (*n* = 23), orbital reconstruction (*n* = 6), no orbital reconstruction (*n* = 17).

^h^ Pain/headache, proptosis, all BOTs ± reconstruction (*n* = 8), orbital reconstruction (*n* = 5), no orbital reconstruction (*n* = 3).

**Table 4:** Postoperative complications and outcomes for intraconal BOTs only

Item	All Intraconal BOTs	Orbital reconstruction	No orbital reconstruction	*p* value
Short‐term post‐op complications (<2 weeks) (*n* [%])	*n* = 43	*n* = 27	*n* = 16	
Visual acuity defect	2 (4.7)	1 (3.7)	1 (6.3)	0.73
Diplopia	7 (16.3)	2 (7.4)	5 (31.3)	0.04
Cranial nerve palsy	3 (7.0)	2 (7.4)	1 (6.3)	0.88
Epistaxis	3 (7.0)	3 (11.1)	0	0.27
Infection	1 (2.3)	0	1 (6.3)	0.38
Other	5 (11.6)	5 (18.5)	0	n/a
None	26 (60.5)	16 (59.3)	10 (62.5)	0.83
Not specified	1 (2.3)	1 (3.7)	0	n/a
Length of follow up (months, mean ± SD)	*n* = 40	*n* = 25	*n* = 15	
	12.2 ± 9.6	10.1 ± 8.0	25.6 ± 25.5	
Visual acuity (*n* [%])	*n* = 31	*n* = 23	*n* = 8	
Improved/no change	31 (100.0)	23 (100.0)	8 (100.0)	n/a
Worse	0	0	0	
Visual field (*n* [%])	*n* = 21	*n* = 20	*n* = 1	
Improved/no change	21 (100.0)	20 (100.0)	1 (100.0)	n/a
Worse	0	0	0	
Color vision (*n* [%])	*n* = 14	*n* = 14	*n* = 0	
Improved/no change	14 (100.0)	14 (100.0)	0	n/a
Worse	0	0	0	
Diplopia (*n* [%])	*n* = 9	*n* = 2	*n* = 7	
Improved/no change	8 (88.9)	1 (50.0)	7 (100.0)	0.48
Worse	1 (11.1)	1 (50.0)	0	
Proptosis (*n* [%])	*n* = 14	*n* = 4	*n* = 10	
Improved/no change	12 (100.0)	4 (100.0)	10 (100.0)	n/a
Worse	0	0	0	
Enophthalmos (*n* [%])	*n* = 43	*n* = 27	*n* = 16	
	4 (9.3)	3 (11.1)	1 (6.3)	0.60
Pain/headache (*n* [%])	*n* = 4	*n* = 2	*n* = 2	
Improved/no change	2 (100.0)	2 (100.0)	2 (100.0)	n/a
Worse	0	0	0	
Tumor recurrence (*n* [%])	*n* = 43	*n* = 27	*n* = 16	
Yes	1 (2.3)	1 (3.7)	0	1.00
No	25 (58.1)	22 (81.5)	3 (18.8)	
Not specified	17 (39.5)	4 (14.8)	13 (81.3)	

*Note*: NB: all “not specified” removed from statistical analysis.

Abbreviation: BOTs, benign orbital tumors.

Postoperative enophthalmos was notably infrequent. When considering all BOTs, 8.3% of patients demonstrated postoperative enophthalmos, with similar rates among reconstructed and nonreconstructed patients (8.8% and 7.7%, respectively). When considering only patients with preoperative proptosis, rates of postoperative enophthalmos were still generally low regardless of reconstruction status: Of the six patients who presented with preoperative proptosis and who underwent post‐EER reconstruction, 16.7% (*n* = 1) had postoperative enophthalmos. Of the 18 patients who presented with preoperative proptosis for whom reconstruction was foregone, 11.1% (*n* = 2) had postoperative enophthalmos.

## DISCUSSION

As an exclusively endoscopic endonasal technique has been increasingly utilized by surgeons worldwide for resection of benign primary orbital tumors, consideration of postresection orbital reconstruction with the intention of improving clinical outcomes has increased.^8^ Advocates of reconstruction suggest that removal of bone and opening of the periorbita may lead to undesirable postoperative enophthalmos and diplopia, whereas medial orbital wall reconstruction may mitigate morbidity.^7^, ^8^, ^10^ However, according to an expert panel of endoscopic orbital surgeons, only about a quarter of such cases include reconstruction in practice,^3^ highlighting the current debate surrounding the necessity of reconstruction and its implications for outcomes.^9^

Overall, we found that the tendency toward orbital reconstruction following exclusively endoscopic endonasal resection was associated with preoperative factors such as higher CHEER stage for BOTs and preoperative vision compromise (including field defects, color deficits, and decreased acuity). However, the tendency to forego orbital reconstruction was associated with preoperative proptosis and larger average tumor size as well as multiple intraoperative factors, including exposure of orbital fat and EOM. Although there were no statistically significant differences between the reconstruction and non‐reconstruction groups for all BOTs in terms of short‐term postoperative complications or longer‐term outcomes, in patients with intraconal BOTs, there was a higher rate of short‐term (<14 days) postoperative diplopia when reconstruction was foregone than when it was completed. This potential benefit of reconstruction, however, appears short‐term: when assessing longer‐term outcomes (at an average of 2 years postoperatively), there was no difference in the rates of diplopia between reconstructed and nonreconstructed patients.

In the present systematic review and meta‐analysis of EER for BOTs, resection was followed by orbital reconstruction in 56.7% (*n* = 34 cases). Reported rates of orbital reconstruction have ranged greatly from 0% (in a series of 24 tumors, including both benign and malignant etiologies, in which 75% were either biopsies or not identified and only 25% were resected)^10^ to 23.5% (in a systematic review including 17 cases of EER for intraconal orbital cavernous hemangiomas)^12^ and 26.1% (in a series of EER for 26 orbital cavernous hemangiomas).^3^ That the reported rate of reconstruction is higher in the current meta‐analysis is likely related to our exclusion of studies which did not specifically report on whether reconstruction was performed following resection, which resulted in our exclusion of 45 BOTs. If we were to assume that each of these excluded cases did not undergo reconstruction, our rate of reconstruction would be 32.3%, which is more in line with previously reported reconstruction rates following EER for BOTs.^3^, ^12^ The lack of clarity in multiple reports regarding the completion of post‐EER reconstruction highlights the need for greater standardization of outcomes reporting in this field. Efforts to improve standardization of outcomes reporting following EER for BOTs is an area of active research.^4^, ^11^

Following orbital tumor resection, multiple methods are available for medial orbital reconstruction, which may be undertaken in either an immediate or delayed fashion.^8^, ^9^ Reconstructive methods include both alloplastic grafts and autologous tissues^7^ as well as rigid and nonrigid materials.^10^ Reconstructive avenues previously reported have included sinonasal bone/cartilage fragments, calvarial bone grafts, rib bone grafts, free microvascular tissue transfers, fascia lata, nasoseptal flaps, free sinonasal mucosal grafts, and porous polyethylene mesh implants.^3^, ^7^, ^8^, ^9^, ^12^, ^13^ In this study, pedicled flaps were most commonly used (44.1%), followed by free mucosal grafts (32.4%), whereas rigid techniques were much less frequently used (8.8%). While some authors have advocated for immediate rigid reconstruction,^8^ others caution that such an approach can increase the risk of orbital compartment syndrome.^9^ Many authors suggest nonrigid reconstruction techniques are preferable and can safely and effectively be utilized immediately following resection as they accommodate postoperative edema and enable blood and other fluid egress during healing, thereby reducing the risk of ischemia and intraorbital pressure elevation.^9^, ^13^

In assessing preoperative factors, CHEER stage, visual symptoms, orbit position, and tumor size were associated with likelihood of orbital reconstruction. In developing the CHEER staging system, an international, multidisciplinary panel of orbital surgery experts reported their operative practices, including in regard to postresection intraoperative orbital reconstruction. The majority of panelists “never” or “almost never” reconstructed lower CHEER stage tumors (i.e., Stages I–III).^11^ Based on panelist responses, there was a stage‐dependent increase in proclivity toward orbital reconstruction such that the majority of panelists reported performing reconstruction for higher CHEER stages (e.g., the majority of panelists “always” or “almost always” performed reconstruction for Stage VB tumors).^11^ Thus, the current finding that the higher CHEER stage tended to be associated with an increased likelihood of orbital reconstruction is consistent with reported practices by expert surgeons. Furthermore, resection of BOTs with higher CHEER stages generally necessitates increased intraconal dissection, which carries a heightened risk for vision compromise. Indeed, an international, multicenter case series of EER for orbital tumors demonstrated a markedly increased rate of orbital reconstruction following intraconal lesion resection (37.5%) compared to that following extraconal lesion resection (0%).^3^ In the present meta‐analysis we also found a tendency toward orbital reconstruction following EER for BOTs with preoperative vision compromise (i.e., field defects, color deficits, and decreased acuity), which may also be a corollary of higher CHEER stage with increased proximity to neurovascular structures.

The tendency to forego orbital reconstruction following EER for BOTs was associated with preoperative proptosis and larger average tumor size, two factors which are themselves often associated. The resection of larger BOTs would be expected to lead to greater orbital volume loss^9^ and may thus indicate an increased risk of attendant enophthalmos, thereby leading some surgeons to be more likely to reconstruct. Alternatively, in the setting of larger volume BOTs causing preoperative proptosis, the surgeon may be seeking a decompressive effect (potentially for cosmesis) and thus may be more hesitant to reconstruct. Thus, while reconstruction may make sense theoretically, in practice some surgeons may forego reconstruction with potential cosmetic benefits in mind in the setting of benign pathology.

In assessing intraoperative factors, we found that fewer surgical hands, single nostril access, and exposure of orbital fat and EOM were associated with the tendency to forego orbital reconstruction. It is somewhat surprising that reported EOM exposure and orbital fat exposure were associated with a decreased tendency toward intraoperative orbital reconstruction. There is a great deal of attention in the orbital tumor resection literature regarding dissection, exposure, and at times resection of orbital fat as well as exposure, retraction (static or dynamic), and partial resection of extraocular musculature.^2^, ^14^ While such maneuvers may be necessary to approach and complete resection of certain orbital tumors, many authors caution that these maneuvers may lead to enophthalmos, ocular muscle fibrosis, strabismus, diplopia, visual defects, and infection.^15^ It is out of concern for these complications that many authors champion immediate orbital reconstruction, suggesting that reconstruction may prevent such complications.^6^, ^7^, ^8^, ^9^ It is thus notable that the present analysis does not suggest these theoretical concerns have greatly influenced surgical practice patterns regarding orbital reconstruction post‐EER.

Reconstruction is often championed as an avenue to prevent unwanted postoperative enophthalmos and diplopia.^6^, ^7^, ^9^ Yet, for the most part, short‐term postoperative complications, as well as longer‐term outcomes, were similar between the reconstruction and non‐reconstruction groups following EER when considering all BOTs in the present analysis. Whether reconstruction was foregone or completed, the rates of postoperative enophthalmos were generally low when considering all BOTs (7.7% and 8.8%, respectively) or when considering only the patients who presented with preoperative proptosis (11.1% and 16.7%, respectively), who may be at higher risk for enophthalmos following resection of likely larger volume tumors causing proptosis at presentation. These rates of enophthalmos are commensurate to what is reported in the literature, which ranges from 11.8% (in a systematic review in which 23.5% of cases underwent reconstruction)^12^ to 21.7% (in a case series in which 26.1% of cases underwent reconstruction)^3^ despite comparatively lower rates of reconstruction.

Regarding diplopia, there appears to be a potential short‐term benefit of reconstruction. Such a trend was seen when considering all BOTs together, and this finding reached statistical significance when evaluating specifically the intraconal BOTs. In the intraconal BOT group, there was a higher rate of short‐term (within 14 days) postoperative diplopia when reconstruction was foregone compared to when it was completed. Intraconal BOTs are known to pose a greater risk of postoperative morbidity,^5^ including new‐onset diplopia, which is thought to likely be due to a greater degree of EOM instrumentation required for their resection.^3^ The comparatively higher rates of orbital reconstruction following resection of intraconal BOTs (seen in the current meta‐analysis and in prior series)^3^ are often attributed to these heightened technical demands and attendant risks. The current meta‐analysis, however, is not the first to note that postoperative diplopia following EER for BOTs is often transient (only 6.7% of patients reported herein had worse diplopia relative to their preoperative baseline at longer‐term follow‐up).^3^, ^5^ In a prior series of EER for 23 orbital cavernous hemangiomas, 26.1% of patients had worsened diplopia postoperatively, yet all but one of these six patients had resolution of this diplopia within 2–3 months.^3^ Longer‐term resolution of postoperative diplopia does not appear related to reconstruction status. And indeed, in a series of 24 orbital tumor resections, none of whom were reconstructed, Rimmer et al.^10^ identified no new or worsening cases of diplopia postoperatively.^10^

Although the present analysis suggested a potential limited, short‐term benefit of orbital reconstruction following exclusively endoscopic endonasal resection for some BOTs, the majority of outcomes assessed herein did not appear to be affected by reconstruction status. While this may indicate that orbital reconstruction is not a necessity, the general equivalence of outcomes demonstrated by the present analysis could alternatively be due to reconstruction having been well‐selected by surgeons in the reported patients. The current meta‐analysis includes only retrospectively reported cases, and thus the decision to reconstruct was made by the surgeon in each case (rather than by randomization as a part of a trial, for instance). And while the reconstruction is often championed as an avenue to decrease postoperative morbidity,^6^, ^8^, ^9^ many authors acknowledge that not all orbital tumors require reconstruction as a part of their surgical management.^7^, ^10^, ^12^ Reconstruction may not be needed following an orbital tumor biopsy or resection of smaller lesions.^10^ Tumors requiring more complex dissection (often reflected by a higher CHEER stage) or resulting in greater disruption of orbital septa and attendant intranasal fat herniation may benefit from post‐resection reconstruction.^10^, ^11^ Ultimately, management decisions, including the decision to complete orbital reconstruction, must be made by the individual surgeon and ought to be tailored to the specific patient and orbital tumor being addressed.^5^, ^11^

The limitations of the current study include the retrospective nature of this review, the quality of data synthesized and reported, and the modest sample sizes for many variables studied—all of which limit our ability to make definitive conclusions from these data. Furthermore, there may be a publication bias toward positive clinical outcomes, which may obscure the accuracy of outcome inferences. As endoscopic endonasal surgical techniques continue to advance, future literature regarding EER of BOTs and reconstructive considerations would benefit greatly from high‐quality, standardized outcomes reporting. Larger, prospective series are needed to further delineate clinical outcomes relative to intraoperative decision‐making and to create evidence‐based protocols for endoscopic management of orbital tumors.

## CONCLUSION

As exclusively endoscopic endonasal treatment of benign primary orbital tumors has become more widespread, high‐quality outcomes data are lacking regarding the decision of when and how to reconstruct following resection.^4^, ^11^ In the present systematic review and meta‐analysis, we found that the tendency toward orbital reconstruction was associated with various preoperative (e.g., CHEER stage, visual symptomatology, orbit position, and tumor size) and intra‐operative factors (e.g., orbital fat and EOM exposure). Although the present analysis suggests a potential limited, short‐term benefit of orbital reconstruction following exclusively endoscopic endonasal resection for benign primary orbital tumors (most markedly for intraconal BOTs in regard to short‐term postoperative diplopia), the majority of outcomes assessed herein did not appear to be affected by reconstruction status. This general equivalence of outcomes may suggest that orbital reconstruction is not a necessity in these cases or alternatively that the decision to reconstruct was well‐selected by surgeons in the reported cases included in this systematic review and meta‐analysis.

## CONFLICT OF INTERESTS

The authors declare that there are no conflict of interests.

## ETHICS STATEMENT

This systematic review and meta‐analysis was considered exempt from IRB review.

## AUTHOR CONTRIBUTIONS

**Ashton E. Lehmann**: conceptualization, data curation, supervision, writing–original draft/reviewing/editing. **Manuela von Sneidern**: data curation, methodology, writing–original draft. **Sarek A. Shen**: formal analysis, writing–review/editing. **Ian M. Humphreys**: writing–review/editing. **Waleed M. Abuzeid**: writing–review/editing. **Aria Jafari**: conceptualization, supervision, writing–original draft/reviewing/editing.

## Data Availability

Data are available via source literature, for which all citations are included in the manuscript.
